# Expectations and Outcomes From Glucagon-Like Peptide-1 Receptor Agonists As Adjunct Treatment for Type 1 Diabetes – Case Presentations

**DOI:** 10.1177/19322968241305641

**Published:** 2024-12-21

**Authors:** Sujatha Seetharaman, Eda Cengiz

**Affiliations:** 1 Division of Pediatric Endocrinology and Diabetes, Department of Pediatrics, University of California, San Francisco, San Francisco, CA, USA

**Keywords:** type 1 diabetes, liraglutide, semaglutide, tirzepatide, glucagon-like peptide receptor agonists

## Abstract

**Background:**

Type 1 diabetes (T1D) is characterized by the autoimmune destruction of pancreatic beta cells, leading to lifelong insulin dependence. Despite advancements in insulin therapies and glucose monitoring, maintaining optimal blood glucose control remains challenging with common issues like weight gain and glucose variability. Glucagon-like peptide 1 receptor agonists (GLP-1 RAs), approved for type 2 diabetes and obesity, are being explored off-label for T1D.

**Case Report:**

This case series investigates the effectiveness of GLP-1 RAs, mainly semaglutide and tirzepatide, as an adjunct therapy to insulin in adolescents and young adults (AYA) with T1D, in a single center, providing real-world insights and highlighting practical issues.

**Discussion:**

Most patients had obesity, consistent with typical indication for use in AYA. Common gastrointestinal side effects improved with dose titration, but careful monitoring is needed for persistent symptoms. One patient developed an eating disorder, underscoring the need for vigilance. Insurance and medication shortage issues impacted treatment continuity, highlighting the need for better support. Glycemic parameters improved in most patients, with weight reduction in several patients with obesity, and no reported diabetic ketoacidosis.

**Conclusions:**

GLP-1 RAs can be a beneficial adjunct therapy in T1D, improving glycemic control, reducing insulin needs, and supporting weight management, while potentially preventing long-term cardiovascular and renal complications.

## Introduction

Type 1 diabetes (T1D) is a chronic condition characterized by the autoimmune destruction of the pancreatic beta cells that produce insulin. Consequently, individuals with this condition have lifelong dependence on insulin through multiple daily injections as a continuous infusion via pump. However, despite progress in insulin formulations, delivery systems, and continuous glucose monitoring, achieving precise control of blood sugars remains difficult.^1,2^ Further, challenges such as hypoglycemia, weight gain, and glucose variability are common, and they prevent providing optimal glucose control.

Under normal physiological conditions, glucose levels are regulated by the hormone glucagon-like peptide-1 (GLP-1) which is produced by the L-cells in the small intestine and distal colon.^3^ GLP-1 binds to receptors located in various organs including the islets of the pancreas, lungs, hypothalamus, stomach, heart, and kidneys.^4^ This hormone plays a crucial role as an incretin by promoting insulin secretion from the pancreatic beta cells and suppressing glucagon release from the alpha cells.^3,5-7^ Additionally, GLP-1 helps to control appetite by influencing the brain and slows down gastric emptying,^8^ leading to a feeling of satiety.^3^ Native GLP-1 has a very short half-life of less than 2 minutes, making it unsuitable for therapeutic use. This rapid breakdown, primarily by dipeptidyl peptidase-4 (DPP-4), means only a small fraction reaches circulation.^9^ To address this, synthetic GLP-1 receptor agonists (GLP-1 RAs) that are variably resistant to degradation by DPP-4 and have longer half-lives have been developed, enabling a more effective and sustained GLP-1 activity. These GLP-1 RAs have recently gained approval for treating type 2 diabetes (T2D) and obesity in both adults and adolescents. Studies involving individuals with T1D have shown promising results, such as reduced insulin needs, minimized glucose variability, weight loss, and better post-meal glucose control.^10-18^ In addition, GLP-1 RAs have been shown to improve cardiovascular and renal outcomes in patients with T2D^19,20^ and T1D.^21^ Although not FDA approved for T1D, several healthcare providers prescribe GLP-1 RAs off-label, especially for patients facing insulin resistance and obesity. This case series investigates the effectiveness and outcomes of GLP-1 RAs as an adjunct therapy to insulin in adolescents and young adults with T1D.

## Methods

This case series was conducted at a single institution and included adolescents and young adults (0-35 years) with T1D who received GLP-1 RAs in addition to standard insulin therapy. Providers were asked about their prescriptions of liraglutide, semaglutide, or tirzepatide. Participants were selected based on clinical need, particularly sub-optimal glycemic control or concurrent obesity. Data on baseline demographics, diabetes duration, changes in insulin dosage, body weight, HbA1c, and incidence of hypoglycemic events before and after treatment with GLP-1 RAs were collected from medical records and insulin pump/Dexcom data. Some data, such as weight and body mass index (BMI), were missing due to telehealth visits.

## Case Discussion

### Case 1

A 26-year-old female diagnosed with T1D for 16 years, managed with the t: slim insulin pump and Dexcom CGM, who also had obesity (BMI 35.2 kg/m^2^, weight 225 lbs.). Her A1c was in target at 6.6% to begin with for glycemic management. In July 2023, she was prescribed Ozempic^®^ with a starting dose of 0.25 mg per week for weight management. Her Ozempic^®^ dose was increased to 2 mg per week in September 2023. However, she had to stop the medication in January 2024 since her insurance denied it. We prescribed Zepbound^®^ after that but was also denied by her insurance. By this time, she had lost around 45 lbs., and she was fine with holding off the medication at this time. Her pump downloads from May 2023 and January 2024 did not demonstrate a change in time in range (TIR) but she had a decrease in her total insulin dose, with no increase in severe episodes of hypoglycemia, with continued maintenance of good glycemic control (Table 1). No significant side effects reported by patient while on the GLP-1 RA treatment.

**Table 1.** Glycemic and Anthropometric Characteristics for Case 1.

Variable	May 2023	Jan 2024
Time in range (TIR)	78%	77%
Time in hypoglycemic range (54-70 mg/dL)	1%	0%
Time in severe hypoglycemic range (<54 mg/dL)	0%	0%
Time in hyperglycemic range (180-250 mg/dL)	18%	19%
Time in hyperglycemic range (>250 mg/dL)	3%	4%
Average glucose (mg/dL)	148	147
Glucose management indicator (GMI)	6.8%	6.8%
Glucose variability	30.3%	33.1%
Average basal insulin/day (units, %)	32.5 (52%)	29.9 (73%)
Average bolus insulin/day (units, %)	29.8 (48%)	11.1 (27%)
Average insulin dose/day (units)	62.3	41.1
Average insulin dose/kg/day (units)	0.6	0.5
Weight (kg)	102.3	81.8
Body mass index (BMI) (kg/m^2^)	35.2	28.3
HbA1c (venous)	6.6%	6.8%

### Case 2

An 18-year-old male with T1D for 12 years, also with obesity (BMI: 40.2 kg/m^2^), is managed with an Omni pod insulin pump and Dexcom CGM. He was prescribed Saxenda^®^ at a starting dose of 0.6 mg in March 2023 for obesity but developed headaches, nausea, and vomiting by the third day. Despite some weight loss, he stopped Saxenda^®^ after a month due to persistent nausea and inability to eat. In May 2023, he was prescribed Wegovy^®^ with a starting dose of 0.25 mg weekly and remained on lower doses due to medication shortages. Patient reported decreased portion sizes, feeling fuller faster, and started experiencing mild weight loss, although there was no change in insulin requirements. He stopped Wegovy^®^ in the summer of 2023 due to ongoing medication shortages.

In December 2023, he was prescribed Zepbound^®^ at a starting dose of 2.5 mg weekly. After a few initial weeks of nausea, his symptoms improved with use of GasX and Pepcid prn. His average blood glucose levels decreased, portion sizes became smaller and lost a few pounds. In Jan 2024, his Zepbound^®^ dose was increased to 5 mg weekly. By May 2024, we increased his Zepbound^®^ dose to 7.5 mg once a week. He also began regular exercise and healthier diet choices. By May 2024, he had lost around 3-4 lbs., and his BMI decreased slightly to 39.2 kg/m^2^. His TIR improved, average glucose level improved, total insulin dose reduced, slight decrease in CV, although HbA1c slightly increased by point of care test (POCT) (Table 2).

**Table 2.** Glycemic Characteristics for Case 2.

Variable	June 2023	May 2024
Time in range (TIR)	41%	54%
Time in hypoglycemic range (54-70 mg/dL)	0%	0%
Time in severe hypoglycemic range (< 54 mg/dL)	0%	0%
Time in hyperglycemic range (180-250 mg/dL)	27%	27%
Time in hyperglycemic range (> 250 mg/dL)	32%	19%
Average glucose (mg/dL)	217	190
Glucose management indicator (GMI)	8.5	NA
Glucose variability	NA	NA
Average basal insulin/day (units)	51.7 (54%)	40.1 (56%)
Average bolus insulin/day (units)	43.9 (46%)	31.8 (44%)
Average insulin dose/day (units)	95.6	72
Average insulin dose/kg/day (units)	0.8	0.6
Weight (kg)	124.7	122
Body mass index (BMI) (kg/m^2^)	40.2	39.2
HbA1c (POCT)	7.9 %	8.2%

Abbreviations: NA: Not available; POCT: Point of care test.

### Case 3

An 18-year-old female with T1D for five years, managed with Omnipod insulin pump and Dexcom CGM, had a BMI of 22.9 kg/m^2^ (weight 60.9 kg, 134 lbs.). She was prescribed Ozempic^®^ at a starting dose of 0.25 mg weekly, in September 2022 as an adjunct therapy for post-prandial hyperglycemia and appetite regulation. Prior to that, she used inhaled insulin (Afreeza^®^) at least once daily to help resolve post-prandial hyperglycemia, which was later denied by her insurance. By January 2023, due to pharmacy shortages and concerns about diarrhea, weight loss, and mild nausea, her family reduced her Ozempic^®^ dose from 0.25 to 0.2 mg weekly. She was referred to a GI clinic for diarrhea and weight loss, who did a thorough evaluation and diagnosed her with isomaltase deficiency. She was subsequently started on digestive enzymes that helped with her diarrhea. Her weight was 63.2 kg (137 lbs.) in Sept 2022, 57.4 kg (128 lbs.) in Jan 2023 and 58.9 kg (130 lbs.) in May 2024. Her BMI decreased from 22.9 in October 2022 to 21.6 in May 2024. Despite these challenges, her episodes of severe hypoglycemia decreased, her CV decreased, and she continued to maintain good glycemic control (Table 3). In October 2023, her mother requested us to increase the dose of Ozempic^®^ because it had helped stabilize her blood glucose levels overnight, and she has continued Ozempic^®^ 2 mg weekly since then and is tolerating it fine.

**Table 3.** Glycemic Characteristics for Case 3.

Variable	October 2022	May 2023
Time in range (TIR)	76%	81%
Time in hypoglycemic range (54-70 mg/dL)	5%	7%
Time in severe hypoglycemic range (< 54 mg/dL)	5%	3%
Time in hyperglycemic range (180-250 mg/dL)	11%	8%
Time in hyperglycemic range (> 250 mg/dL)	3%	1%
Average glucose (mg/dL)	121	117
Glucose management indicator (GMI)	6.2%	6.1%
Glucose variability	44%	38%
Average basal insulin/day (units, %)	17.6 (38%)	15.1 (33%)
Average bolus insulin/day (units, %)	28.1 (62%)	31.1 (67%)
Average insulin dose/day (units)	45.7	46.2
Average insulin dose/kg/day (units)	0.8	0.8
Weight (kg)	60.9	57.4
Body mass index (BMI) (kg/m^2^)	22.9	21.6
HbA1c (POCT)	6 % (Jan 2022)	NA

Abbreviations: NA: Not available; POCT: Point of care test.

### Case 4

A 14-year-old male with T1D for 6 years, managed with a t:slim insulin pump and decom CGM, also with obesity (BMI 30 kg/m^2^, weight 84.4 kg) and insulin resistance. He was prescribed Ozempic^®^ at a starting dose of 0.25 mg weekly, in May 2023 for obesity. During his visit in July 2023, he had lost 10 lbs. while he was also making lifestyle changes. He denied any side effects with the Ozempic^®^. His A1c decreased to 7% and his insulin doses started to decrease as his blood glucose levels started to improve (Table 4)

**Table 4.** Glycemic Characteristics for Case 4.

Variable	May 2023	Sept 2023
Time in range (TIR)	46%	73%
Time in hypoglycemic range (54-70 mg/dL)	0.03%	1%
Time in severe hypoglycemic range (< 54 mg/dL)	0.1%	0.2%
Time in hyperglycemic range (180-250 mg/dL)	27%	16%
Time in hyperglycemic range (> 250 mg/dL)	26%	10%
Average glucose (mg/dL)	202	152
Glucose management indicator (GMI)	8.1%	7%
Glucose variability	36%	41%
Average basal insulin/day (units, %)	68.1 (51%)	38.7 (67%)
Average bolus insulin/day (units, %)	64.8 (49%)	18.8 (33%)
Average insulin dose/day (units)	132.9	57.5
Average insulin dose/kg/day (units)	1.57	0.9
Weight (kg)	84.4	64.4
Body mass index (BMI) (kg/m^2^)	30	23
HbA1c (POCT)	7.8 %	5.6%

Abbreviation: POCT: Point of care test.

However, in September 2023, we stopped his Ozempic^®^ due to rapid weight loss of 44 lbs. in 3.5 months. He was restricting his oral intake to 800 calories per day and running 30 minutes to an hour daily. He endorsed that he had a goal of losing 50 lbs. Therefore, we referred him to the adolescent eating disorder clinic for a comprehensive evaluation.

### Case 5

A 13-year-old male with T1D for 3.5 years and obesity BMI (34.8 kg/m^2^, weight 83.3 kg, 183.3 lbs.), initially managed with multiple daily insulin injections and a Dexcom CGM. His medical history included conductive hearing loss, asthma, developmental delay, and long-standing hemoglobin A1c in the 12% range, which improved to 8.4% in May 2024. He was started on a t: slim insulin pump and Dexcom CGM in July 2023. He was prescribed Wegovy^®^ at a starting dose of 0.25 mg weekly, in March 2023 for obesity but discontinued after 8 doses due to vomiting, dizziness, and diarrhea. In December 2023, an attempt to restart Wegovy^®^ was unsuccessful due to medication shortage. Subsequently, he was started on Zepbound^®^ at a starting dose of 2.5 mg weekly in February 2024, with the dose gradually increased to 5 mg and then 7.5 mg weekly. However, by May 2024, Zepbound^®^ was also on back order, and the medication was stopped. His weight increased significantly, reaching 117.5 kg (258.5 lbs.; BMI 47.2 kg/m^2^) in April 2024. He was then referred to the weight management clinic for a possible bariatric surgery evaluation. His glycemic parameters showed a decrease in TIR, an increase in average glucose level, and an increase in GMI, although his A1c by POCT was lower than in July 2023 (Table 5).

**Table 5.** Glycemic Characteristics for Case 5.

Variable	February 2024	April 2024
Time in range (TIR)	38%	13%
Time in hypoglycemic range (54-70 mg/dL)	0%	0%
Time in severe hypoglycemic range (< 54 mg/dL)	0%	0%
Time in hyperglycemic range (180-250 mg/dL)	24%	12%
Time in hyperglycemic range (> 250 mg/dL)	38%	74%
Average glucose (mg/dL)	220	309
Glucose management indicator (GMI)	8.6%	10.7%
Glucose variability	40.7%	28.8%
Average basal insulin/day (units, %)	97.1 (81%)	92.0 (75%)
Average bolus insulin/day (units, %)	22.5 (19%)	30.6 (25%)
Average insulin dose/day (units)	119.7	122.6
Average insulin dose/kg/day (units)	1.1	1.04
Weight (kg)	112.6	117.5
Body mass index (BMI) (kg/m^2^)	45.2	47.2
HbA1c (POCT)	8%	8.8%

Abbreviation: POCT: Point of care test.

### Case 6

A 26-year-old female with T1D for 23 years, managed with a t:slim insulin pump and Dexcom CGM, also with obesity (BMI: 33.6 kg/m^2^; weight: 80.4 kg; 177 lbs.). She was prescribed Wegovy^®^ at a starting dose of 0/25 mg weekly, in March 2023 and tolerated it well without any side effects. She reported feeling fuller after meals and decreased appetite. In June 2023, Wegovy^®^ was out of stock and an attempt to switch to Ozempic^®^ was made but it was denied by her insurance. Wegovy^®^ became available again a few weeks later and she continued to experience reduced appetite, increased fullness, and occasional nausea without vomiting. She was prescribed Zofran as needed for nausea. Wegovy^®^ dose was titrated to 1 mg weekly but she was not consistently taking it, so we kept her on the same dose.

In November 2023, Wegovy^®^ went out of stock again but became available in December 2023. She restarted Wegovy^®^ at 0.5 mg weekly. She started to have a decrease in her insulin doses and improved glycemic control. Her weight had decreased to 75.8 kg, 167 lbs. However, by March 2024, her weight increased to 78.1 kg, 172 lbs. Her dose of Wegovy^®^ was titrated up to 1 mg weekly. Her TIR, average glucose level, GMI, and CV have all improved, and insulin doses have reduced since starting GLP-1 RA therapy, as seen in Table 6.

**Table 6.** Glycemic Characteristics for Case 6.

Variable	February 2023	March 2024
Time in range (TIR)	59%	70%
Time in hypoglycemic range (54-70 mg/dL)	1%	4%
Time in severe hypoglycemic range (<54 mg/dL)	0.2%	1%
Time in hyperglycemic range (180-250 mg/dL)	26%	19%
Time in hyperglycemic range (>250 mg/dL)	13%	6%
Average glucose (mg/dL)	181	146
Glucose management indicator (GMI)	7.7%	6.8%
Glucose variability	42%	38.2%
Average basal insulin/day (units, %)	40.2 (62%)	33.6 (67%)
Average bolus insulin/day (units, %)	24.9 (38%)	16.9 (33%)
Average insulin dose/day (units)	65.2	50.5
Average bolus insulin/kg/day (units)	0.8	0.6
Weight (kg)	80.4	78.1
Body mass index (BMI) (kg/m^2^)	33.6	32.5
HbA1c (POCT)	7.8 %	6.8%

Abbreviation: POCT: Point of care test.

### Case 7

A 23-year-old female with T1D for 12 years, managed with a t: slim insulin pump and Dexcom CGM, who also had obesity (weight 93 kg, 205 lbs., BMI 30.3 kg/m^2^), weight 93 kg, 205 lbs. She was prescribed Ozempic^®^ at a starting dose of 0.25 mg weekly, in January 2023 and tolerated it well. She reported decreased appetite, weight loss, and improved glucose levels, without experiencing nausea or other gastrointestinal symptoms. By December 2023, her weight had decreased to 79.4 kg (175 lbs.). She was satisfied with her results at 1 mg weekly and therefore we maintained her on this dose for several months. In May 2024, we increased her dose to 2 mg weekly. She showed improvements in her TIR, GMI, A1c, and average glucose levels as seen below (Table 7).

**Table 7.** Glycemic Characteristics for Case 7.

Variable	January 2023	May 2024
Time in range (TIR)	69%	78%
Time in hypoglycemic range (54-70 mg/dL)	0.2%	2%
Time in severe hypoglycemic range (< 54 mg/dL)	0%	1%
Time in hyperglycemic range (180-250 mg/dL)	21%	15%
Time in hyperglycemic range (> 250 mg/dL)	10%	3%
Average glucose (mg/dL)	166	136
Glucose management indicator (GMI)	7.3%	6.6%
Glucose variability	35%	37%
Average basal insulin/day (units, %)	40.2 (62%)	33.6 (67%)
Average bolus insulin/day (units, %)	24.9 (38%)	16.9 (33%)
Average insulin dose/day (units)	65.2	57.6
Average bolus insulin/kg/day (units)	0.7	0.7
Weight (kg)	93	77.1
Body mass index (BMI) (kg/m^2^)	30.3	25.1
HbA1c (POCT)	7.8 %	6.8%

Abbreviation: POCT: Point of care test.

### Case 8

A 14-year-old female with a 3-year history of T1D, managed with an Omnipod insulin pump and Dexcom CGM, presented with a history of polycystic ovary syndrome and obesity (BMI 32.2 kg/m^2^, weight 83.6 kg).

In March 2024, she was prescribed Wegovy^®^ at a starting dose of 0.25 mg weekly, for weight management. However, due to supply shortages, she was switched to Ozempic^®^ 0.25 mg weekly in April 2024. After the first two doses of Ozempic^®^, she experienced mild side effects, including slight difficulty in staying asleep due to discomfort at the injection site. She reported no change in appetite or nausea. She, however, reported an increase in post-prandial hypoglycemia, managed with simple carbs and not severe with no glucagon use. Despite these mild side effects, she continued with the treatment. Therefore, we continued the same dose of 0.25 mg of Ozempic^®^ weekly with a plan to increase the dose once hypoglycemic episodes resolved. We also weakened her insulin-carbohydrate ratio. Although her HbA1c decreased from 8 to 7.4% after 3 months, there were no significant episodes of severe hypoglycemia or change in other glycemic parameters, as shown in Table 8.

**Table 8.** Glycemic Characteristics for Case 8.

Variable	February 2024	July 2024
Time in range (TIR)	60%	58%
Time in hypoglycemic range (54-70 mg/dL)	2%	0%
Time in severe hypoglycemic range (< 54 mg/dL)	0%	0%
Time in hyperglycemic range (180-250 mg/dL)	25%	32%
Time in hyperglycemic range (> 250 mg/dL)	11%	11%
Average glucose (mg/dL)	169	177
Glucose management indicator (GMI)	7.3%	7.4%
Glucose variability	35.1%	31.5%
Average basal insulin/day (units, %)	30.4 (43%)	46 (67%)
Average bolus insulin/day (units, %)	40.1 (57%)	22.4 (33%)
Average insulin dose/day (units)	70.5	68.9
Average bolus insulin/kg/day (units)	0.8	0.8
Weight (kg)	83.6	81.9
Body mass index (BMI) (kg/m^2^)	32.2	31.8
HbA1c (POCT)	8%	NA

Abbreviations: NA: Not available; POCT: Point of care test.

## Discussion

This case series is the first to demonstrate outcomes from several adolescents and young adults with T1D who are on GLP-1 RAs, providing valuable real-world experience with these medications and highlighting practical issues associated with their use. The GLP-1 RAs used by patients in this case series included semaglutide, which is FDA approved as Wegovy^®^ for both adult and adolescent obesity and as Ozempic^®^ for type 2 diabetes in adults, and tirzepatide, which is FDA approved as Zepbound^®^ for adults with obesity.

Most patients included in the case series had obesity, consistent with the typical off-label use of GLP-1 RAs in T1D patients who struggle with obesity. Only one patient (case 3) did not have obesity, offering unique insights into the effects of GLP-1 RAs in a different subset of T1D patients. This diversity in patient demographics underscores the potential versatility of GLP-1 RAs in managing T1D beyond indications of obesity.

A significant number of patients experienced gastrointestinal side effects, such as nausea, vomiting and diarrhea, even at low doses of GLP-1 RAs. These symptoms generally improved after a few weeks with some patients managing them with medications like Zofran or Pepcid as needed. Gradual dose titration helped minimize these common GI issues. However, one patient (case 3) with a normal BMI developed persistent diarrhea and weight loss. Further investigation revealed an isomaltase deficiency, highlighting the importance of careful monitoring and differential diagnosis when side effects persist. This case exemplifies the necessity for healthcare providers to remain vigilant for underlying conditions that may mimic or exacerbate medication side effects. Also, due to their impact on gastric emptying, patients with gastroparesis or inflammatory bowel diseases should avoid GLP-1 RAs.

While most patients experienced mild to moderate weight loss and some did not (case 5), one patient (case 4) developed significant rapid weight loss and restrictive eating behaviors, raising concerns about a potential eating disorder and prompting us to discontinue his GLP-1 RA. This patient was subsequently referred to the adolescent clinic for a comprehensive evaluation of an eating disorder. This scenario highlights the critical need for close monitoring of disordered eating patterns in patients on GLP-1 RAs, particularly in those already at risk for such behaviors. The balance between therapeutic benefits and potential psychological impacts must be carefully managed.

Insurance and medication shortage issues posed significant barriers for several patients. Some had to halt their medications, take breaks in their treatment courses, or switch the type of GLP-1 RA due to lack of coverage or backorders at pharmacies. This inconsistency in treatment can impact the overall effectiveness and continuity of care, emphasizing the urgent need for improved supply and better insurance support for the use of GLP-1 RAs. The financial burden on patients cannot be overlooked, and systemic changes are required to ensure that innovative treatments are accessible to all who might benefit from them.

Several glycemic parameters improved in most patients, with notable improvements in TIR, CV, average glucose levels, and A1c levels. Some studies have reported hypoglycemia and ketosis with liraglutide, necessitating close monitoring.^10,11^ In this case series, one patient (case 8) experienced an increase in hypoglycemic episodes, necessitating a decrease in insulin dose. Importantly, there were no episodes of diabetic ketoacidosis reported among the patients, indicating that GLP-1 RAs can be safely used in this population. These findings suggest that GLP-1 RAs can be a beneficial adjunct therapy with insulin for improving glycemic control in T1D patients. Furthermore, some studies have reported a potential risk of depression and suicidal ideation, which require screening before and during the treatment.^22,23^ However, no patients in this case series exhibited significant depression, suicide risk, or mood issues, indicating that with careful monitoring, these risks can be managed effectively.

Incorporating GLP-RAs with insulin therapy for patients with T1D shows potential in improving glycemic control, reducing insulin needs, and supporting weight management. Ongoing trials suggest that this combination could also prevent cardiovascular and renal complications, improving long-term health outcomes and quality of life for T1D patients.
